# A Virus-Derived Stacked RNAi Construct Confers Robust Resistance to Cassava Brown Streak Disease

**DOI:** 10.3389/fpls.2016.02052

**Published:** 2017-01-18

**Authors:** Getu Beyene, Raj Deepika Chauhan, Muhammad Ilyas, Henry Wagaba, Claude M. Fauquet, Douglas Miano, Titus Alicai, Nigel J. Taylor

**Affiliations:** ^1^Institute for International Crop Improvement, Donald Danforth Plant Science Center, St. LouisMO, USA; ^2^School of Plant Sciences, University of Arizona, TucsonAZ, USA; ^3^National Crops Resources Research InstituteKampala, Uganda; ^4^Global Cassava Partnership for the 21st Century, International Center for Tropical AgricultureCali, Colombia; ^5^Department of Plant Science and Crop Protection, University of NairobiNairobi, Kenya

**Keywords:** cassava brown streak disease (CBSD), resistance to CBSD, RNAi, TME 204, transgenic, siRNA, CBSV, UCBSV

## Abstract

Cassava brown streak disease (CBSD) threatens food and economic security for smallholder farmers throughout East and Central Africa, and poses a threat to cassava production in West Africa. CBSD is caused by two whitefly-transmitted virus species: *Cassava brown streak virus* (CBSV) and *Ugandan cassava brown streak virus* (UCBSV) (Genus: *Ipomovirus*, Family *Potyviridae*). Although varying levels of tolerance have been achieved through conventional breeding, to date, effective resistance to CBSD within East African cassava germplasm has not been identified. RNAi technology was utilized to integrate CBSD resistance into the Ugandan farmer-preferred cassava cultivar TME 204. Transgenic plant lines were generated expressing an inverted repeat construct (p5001) derived from coat-protein (CP) sequences of CBSV and UCBSV fused in tandem. Northern blots using probes specific for each CP sequence were performed to characterize 169 independent transgenic lines for accumulation of CP-derived siRNAs. Transgenic plant lines accumulating low, medium and high levels of siRNAs were bud graft challenged with the virulent CBSV Naliendele isolate alone or in combination with UCBSV. Resistance to CBSD in the greenhouse directly correlated to levels of CP-derived siRNAs as determined by visual assessment of leaf and storage root symptoms, and RT-PCR diagnosis for presence of the pathogens. Low expressing lines were found to be susceptible to CBSV and UCBSV, while medium to high accumulating plant lines were resistant to both virus species. Absence of detectable virus in the best performing p5001 transgenic lines was further confirmed by back-inoculation via sap or graft challenge to CBSD susceptible *Nicotiana benthamiana* and cassava cultivar 60444, respectively. Data presented shows robust resistance of transgenic p5001 TME 204 lines to both CBSV and UCBSV under greenhouse conditions. Levels of resistance correlated directly with levels of transgene derived siRNA expression such that the latter can be used as predictor of resistance to CBSD.

## Introduction

Cassava production in sub-Saharan Africa is constrained by two important viral diseases: cassava mosaic disease (CMD) and cassava brown streak disease (CBSD). Distribution of CBSD is presently restricted mostly to eastern and central Africa. However, CBSD is spreading, and now presents a significant threat to cassava production in West Africa, including Nigeria, the world’s largest cassava producing country (Legg et al., 2014; Patil et al., 2015). CBSD is caused by *Cassava brown streak virus* (CBSV) and *Ugandan cassava brown streak virus* (UCBSV) (Monger et al., 2001; Mbanzibwa et al., 2009; Winter et al., 2010), both of which are positive sense single stranded RNA viruses belonging to the family *Potyviridae*, genus *Ipomovirus*. Both viruses are transmitted by the cassava whitefly, *Bemisia tabaci* (Maruthi et al., 2005). Yield loss due to CBSD on susceptible cultivars can reach up to 70% (Hillocks et al., 2001), with recent data reporting losses valuing up to US$75 million in eight eastern and central African countries (Patil et al., 2015). CBSD causes symptoms on leaves and stems that act to weaken the plant over successive vegetative propagation cycles. Its major impact, however, is production of necrotic lesions within the storage roots. This renders affected roots unfit for consumption or sale in the market, thereby impacting both food and economic security for smallholder farmers in regions where CBSD is present. Unlike CMD, where sources of inherent resistance have been identified (Akano et al., 2002; Lokko et al., 2005; Okogbenin et al., 2012; Rabbi et al., 2014), most farmer-preferred cassava cultivars are susceptible, or highly susceptible, to CBSD. Breeding programs targeting introgression of CBSD resistance from resistant/tolerant cultivars are ongoing (Kawuki et al., 2016), but such efforts are time consuming and face challenges in combining effective resistance to CBSD and CMD with good storage root quality and other attributes preferred by farmers and consumers. To date, the best varieties deployed to control CBSD are tolerant to the disease and rapidly degenerate under high virus inoculum pressure after 2–3 cropping cycles.

Application of transgenic RNAi technology has been successful in generating resistance to plant viral pathogens, especially against RNA-viruses (Collinge et al., 2010; Duan et al., 2012). Previously, we reported control of CBSD in the model plant *Nicotiana benthamiana* (Patil et al., 2011) and in cassava (Yadav et al., 2011; Ogwok et al., 2012; Odipio et al., 2014). These plants were generated to express a hairpin RNAi construct derived from the near full-length coat protein (CP) sequence of UCBSV (UCBSV-CP). The resulting plants of cassava cultivar 60444 were found to be highly resistant to infection by the homologous virus (UCBSV), with some lines also showing significantly elevated resistance to CBSV under field conditions (Ogwok et al., 2012; Odipio et al., 2014). Proof of concept for the control of CBSD by RNAi technology was further strengthened by Vanderschuren et al. (2012), who reported a similar approach utilizing sequences derived from the CP of CBSV (CBSV-CP) to generate resistance in the West African cultivar TME 7. The Virus Resistant Cassava for Africa (VIRCA) project aims to develop, test and deploy CBSD-resistant planting materials to farmers in East Africa (Taylor et al., 2012b). Toward this goal the Ugandan farmer-preferred cassava cultivar TME 204 was genetically modified with an improved RNAi construct designed to provide high levels of resistance to both CBSV and UCBSV. We report here characterization of TME 204 plant lines transgenic for an inverted repeat construct derived from the CP sequences of *Ugandan cassava brown streak virus* (UCBSV-CP) and *Cassava brown streak virus* (CBSV-CP) fused in tandem (Chauhan et al., 2015). Efficacy of plant lines under greenhouse conditions is demonstrated by high levels of resistance to both pathogens, with resistance directly correlated with expression levels of CP-derived siRNAs. Subsequent performance of TME 204 plants transgenic for the same RNAi construct also demonstrated high levels of resistance to CBSD in multi-location field trials in Uganda and Kenya (Wagaba et al., 2017).

## Materials and Methods

### Production of Fused Coat Protein RNAi Construct and Transgenic Plants

The present study utilized an RNAi construct consisting of an inverted repeat of near full-length CP genes cloned from CBSV isolate Naliendele (CBSV[TZ:Nal3-1:07]) (GenBank HG965221 position 7944-8839) and UCBSV isolate (UCBSV[UG:T04-42:04]) (GenBank HG965222 position 7892-8790). The CP sequences were fused in tandem and the constitutive *Cassava vein mosaic virus* (CsVMV) promoter (Verdaguer et al., 1998) cloned at the 5′-end to generate an expression cassette terminated by the nopalin synthase terminator sequence. The inverted repeat expression cassette was cloned into binary vector p5000 and resulting construct named p5001. p5001 (Beyene et al., 2016) is based on the pCAMBIA2300 vector (GenBank: AF234315) modified by removal of the LacZ open reading frame, insertion of stop codons at the left and right border junctions, removal of unintended open reading frames from the inverted repeats, and insertion of Multi-Left-Border sequences (PureMlb^TM^, Japan Tobacco, Iwata, Shizuoka, Japan) (Kuraya et al., 2004). The p5001 vector was electroporated into *Agrobacterium tumefaciens* strain LBA4404 and used to transform cassava cultivar TME 204. *Agrobacterium*-mediated transformation of friable embryogenic callus (FEC), regeneration of transgenic p5001 TME 204 lines and estimation of T-DNA copy number have been reported by Chauhan et al. (2015). TME 204 plants generated using p5003 (Beyene et al., 2016) and transgenic TME 204 and 60444 lines generated using the p718 construct (Yadav et al., 2011) were used as controls. p5003 and p718 constructs express an inverted repeat of CP sequences derived from CBSV and UCBSV, respectively.

### Detection and Quantification of Small RNAs by Northern Blot Analysis

Transgenic TME 204 plants identified by Dot blot analysis to carry 1–2 copies of the T-DNA (Chauhan et al., 2015) were analyzed for accumulation of transgene CP-derived siRNAs by Northern blotting. Total RNA was extracted from ca. 100 mg of leaf tissue harvested from *in vitro*-grown plantlets using TRIzol Reagent (Ambion, Houston, Texas). Thirty micrograms of total RNA was separated on 15% Criterion^TM^ TBE-Urea Precast Gels (Bio-Rad, Hercules, CA, USA) and transferred to Hybond^TM^ N+ membrane (GE Healthcare Ltd) using a semi-dry trans blotter (Bio-Rad, Hercules, CA, USA). RNA probes for hybridization were prepared using a digoxigenin (DIG) RNA labeling kit with SP6/T7 polymerase according to manufacturer instructions (Roche Applied Science, Indianapolis, IN, USA). Two probes were generated, one each to detect siRNAs specific to CBSV and UCBSV. The partial CP gene sequences of CBSV and UCBSV (hereafter named CBSV-CP and UCBSV-CP, respectively) used to generate the inverted repeat sequence were cloned into the pGEM-T easy vector (Promega, Madison, WI, USA) and utilized as templates for *in vitro* transcription. The probes were hydrolyzed before use. Pre-hybridization of the membrane and hybridization of the probes with membrane bound RNA was performed at 40°C. Membrane washing, detection with CDP-star (Roche Applied Science, Indianapolis, IN, USA) and development of signal using HyperFilm ECL (GE-Healthcare, Buckinghamshire, UK) were performed per manufacturer instructions.

Each RNA sample was run and blotted twice, once for detection of siRNAs specific to the CBSV-CP and the other for UCBSV-CP using respective probes. Signal strength was determined by scanning the blots, followed by quantification using ImageJ software v. 1.50g (Rasband 1997-2015). Each Northern blot included control RNA extracted from plant line 718-01 of cultivar 60444 transgenic for construct p718 that consists of an inverted repeat construct from the CP sequence of UCBSV (Yadav et al., 2011). TME 204 siRNA accumulation data derived from UCBSV-CP was calculated and presented relative to the Northern blot signal strength of 718-01. Similarly, siRNA accumulation data derived from CBSV-CP was calculated and presented relative to 5001-08 (a transgenic line identified to accumulate similar level of UCBSV-CP derived siRNA).

### Greenhouse Challenge of Transgenic Plants with CBSV and UCBSV

p5001 transgenic plants were bud graft inoculated with CBSV and UCBSV according to Wagaba et al. (2013). Plants of cassava cultivar 60444 infected with CBSV isolate Naliendele (CBSV[TZ:Nal3-1:07]), (Mohammed et al., 2012) and UCBSV isolate (UCBSV[UG:T04-42:04], Mbanzibwa et al., 2011) were maintained in the greenhouse and used as the source of inoculum. *In vitro* micropropagated TME 204 plants transgenic for p5001 were transferred to soil (Taylor et al., 2012a) and graft inoculated with one or both virus species at 7–10 weeks of age. Starting 10 days after graft inoculation, CBSD symptoms on leaves and stems were scored visually twice weekly using a 1–5 scale (Hillocks et al., 1996). In some experiments, plant stems were cut back 5–10 weeks after graft inoculation to 5–8 nodes above the graft union. Newly developing leaves and stem tissues were then assessed for CBSD symptom development. For storage root evaluation, plants were harvested at 12–22 weeks post inoculation. Storage roots were washed, the peel removed and each root sliced transversely along its length into 1–2 cm thick sections. Each slice was visually assessed and scored on a scale of 1–5 for presence of CBSD symptoms following root scoring system described by Hillocks et al. (1996).

### RNA Isolation and Detection of the Viruses by RT-PCR

Leaf samples were collected during the course of the experiment and storage root samples at the time of harvest, 12–22 weeks after graft inoculation. Storage root slices were frozen immediately in liquid nitrogen, freeze dried, and ground to a fine powder using a mortar and pestle. Samples of symptomatic and asymptomatic leaves were placed into 2 ml screw cap tubes and immediately frozen in liquid nitrogen. Ground storage root samples and leaf samples were homogenized in a FastPrep-24 machine (MP Biomedical, Solon, OH, USA) within 2 ml screw cap tubes, at settings of 4.0 M/S twice each for 30 s in presence of 700 μl of extraction buffer. For RNA extraction, 100–150 mg fresh weight leaf tissue and approximately 50 mg dry weight storage root tissue was used. RNA isolation was performed following the cetyltrimethylammonium bromide (CTAB) protocol (Doyle and Doyle, 1990). Genomic DNA was removed by DNAse treatment using a TURBO DNA-free^TM^ Kit (Ambion, Houston, Texas) and 2 μg of the resulting total RNA reverse transcribed following the Invitrogen SuperScript^®^ III First-Strand cDNA synthesis system per manufacturer instructions (Invitrogen, Carlsbad, CA, California).

Detection of CBSV and UCBSV was carried out by RT-PCR as described previously by Wagaba et al. (2013), using primers that amplify 344 and 440 bp of the CBSV-CP and UCBSV-CP sequences, respectively (Mohammed et al., 2012). PCR amplification was performed using 1 μl of cDNA template in 20 μl reaction mixture with Phusion High-Fidelity master mix (New England Biolabs, Ipswich, MA, USA). The PCR conditions used were 1 cycle at 98°C for 30 s, followed by 30 cycles of 5 s at 98°C, 10 s at 59°C and 30 s at 72°C, and a final extension for 5 min at 72°C with the reaction held at 4°C. Amplified PCR products were separated on a 1.0% agarose gel containing 0.5 μg/ml ethidium bromide.

### Back-Inoculation to CBSD Susceptible Hosts

Back-inoculation experiments were performed to ascertain whether resistant plant lines were free from one or both viruses. Sap was extracted from graft-challenged transgenic cassava and used to inoculate plants of *N. benthamiana*. Procedures employed for sap inoculation of *N. benthamiana* followed those described previously (Ogwok et al., 2010; Patil et al., 2011). Briefly, 5–6 leaflets from the topmost mature cassava leaves were collected from symptomatic controls and asymptomatic transgenic cassava plants and ground with 0.06 M potassium phosphate extraction buffer in a sterile mortar and pestle. Leaf debris was separated from the sap by squeezing through sterile muslin cloth. Approximately 0.5 ml of clear sap was rubbed with carborundum powder onto the adaxial surface of three fully expanded leaves per *N. benthamiana* plant. For each transgenic cassava line, five clonal replicate plants were selected and inoculum from each plant was used to inoculate ten plants of 2-week-old *N. benthamiana*. After inoculation, CBSD symptom development, manifested as crumpling of leaves, was monitored over a period of 4–6 weeks.

Back-inoculation to susceptible cassava cultivar 60444 was performed by bud grafting from transgenic and non-transgenic plants of TME 204. Buds were excised from TME 204 plants 21 weeks after inoculation with UCBSV and CBSV. Five plants per transgenic line were used as the source of inoculum, with two buds per plant used to back graft onto two 10-week-old plants of cultivar 60444, to generate a total of ten inoculated cultivar 60444 plants per line. CBSD symptom development and severity was recorded on shoot tissues over the subsequent 14 weeks.

## Results

### The CP-Sequences Used for Making the Inverted Repeat Construct

The RNAi inverted repeat construct p5001 was created by generating tandem fusions of near full-length CP gene sequences from CBSV (896 bp: position 7944–8839) Naliendele isolate (CBSV[TZ:Nal3-1:07]) (Mohammed et al., 2012) and UCBSV (899 bp position 7892–8790) from UCBSV isolate (UCBSV[UG:T04-42:04]) (Mbanzibwa et al., 2011). Both isolates have been sequenced and the near complete genome deposited, with GenBank accession numbers HG965221 and HG965222 assigned, respectively, for CBSV and UCBSV. The whole genome of the two virus isolates and the selected CP regions share 71.2 and 73.6% nt sequence identity, respectively. Multiple alignments of the CP sequences used within p5001 show the transgenic CBSV-CP sequence to share 91–94% nt identity with four other CBSV isolates, and UCBSV-CP to share 91.6–98.9% with the seven UCBSV isolates for which genome sequences are available in GenBank (Supplementary Table 1). Similarity of the CP sequences in p5001 to CBSV and UCBSV, cloned and sequenced from the two confined field trial sites, is presented by Wagaba et al. (2017).

### Production of Transgenic Cassava Plants and Detection of CBSV and UCBSV CP-Derived siRNAs

**FIGURE 1:**
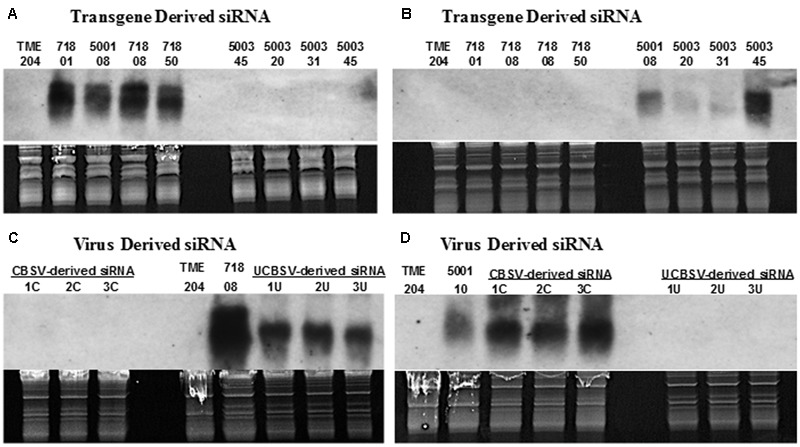
**Northern blot shows virus-specific detection of siRNAs derived from coat-protein (CP) sequences of cassava brown streak viruses.** Transgenic plant lines assayed express inverted repeats of CP genes derived from UCBSV (UCBSV-CP, p718), CBSV (CBSV-CP, p5003) or both UCBSV-CP and CBSV-CP fused in tandem (p5001). For detection of virus specific siRNA, leaf samples were collected from infected, greenhouse-grown cultivar 60444 stock plants. For the detection of transgene-derived siRNA, RNA was extracted from *in vitro* plantlets. **(A,C)** siRNA signals detected with probe specific to CP of UCBSV and **(B,D)** with probe specific to CP of CBSV. Transgenic line 718-01 is generated by transforming cultivar 60444, while lines 718-08 and 718-50 were generated in cultivar TME 204 using the construct p718 (Yadav et al., 2011). All 5003 lines were generated using cultivar TME 204. 1C, 2C, and 3C are wild-type 60444 plants infected with CBSV isolate Naliendele (CBSV[TZ:Nal3-1:07]), while 1U, 2U, and 3U are wild-type 60444 plants infected with UCBSV isolate (UCBSV[UG:T04-42:04]) that have been maintained in the greenhouse.

Approximately 450 independent transgenic cassava plants were recovered after *Agrobacterium*-mediated transformation of cultivar TME 204 (Chauhan et al., 2015). Of these, 418 lines were analyzed by Dot blot to estimate T-DNA copy number, and 169 transgenic low copy (1–2) plant lines further assayed by Northern blotting to determine levels of transgene-derived siRNAs, using probes specific for the respective CP sequences. Northern blots confirmed that the probes produced were specific for CP sequences from the respective viruses. Therefore, the UCBSV-CP probe detected only siRNA derived from plants transgenic for RNAi constructs p5001 and p718 (Patil et al., 2011) and siRNAs produced by non-transgenic plants of cultivar 60444 infected with UCBSV (**Figures 1A,C**). Similarly, probes derived from CBSV-CP detected siRNA in transgenic plants transformed with p5001 and p5003 (plants expressing a CBSV-CP inverted repeat) (Beyene et al., 2016), and from cultivar 60444 plants infected with CBSV isolate Naliendele (**Figures 1B,D**).

**FIGURE 2:**
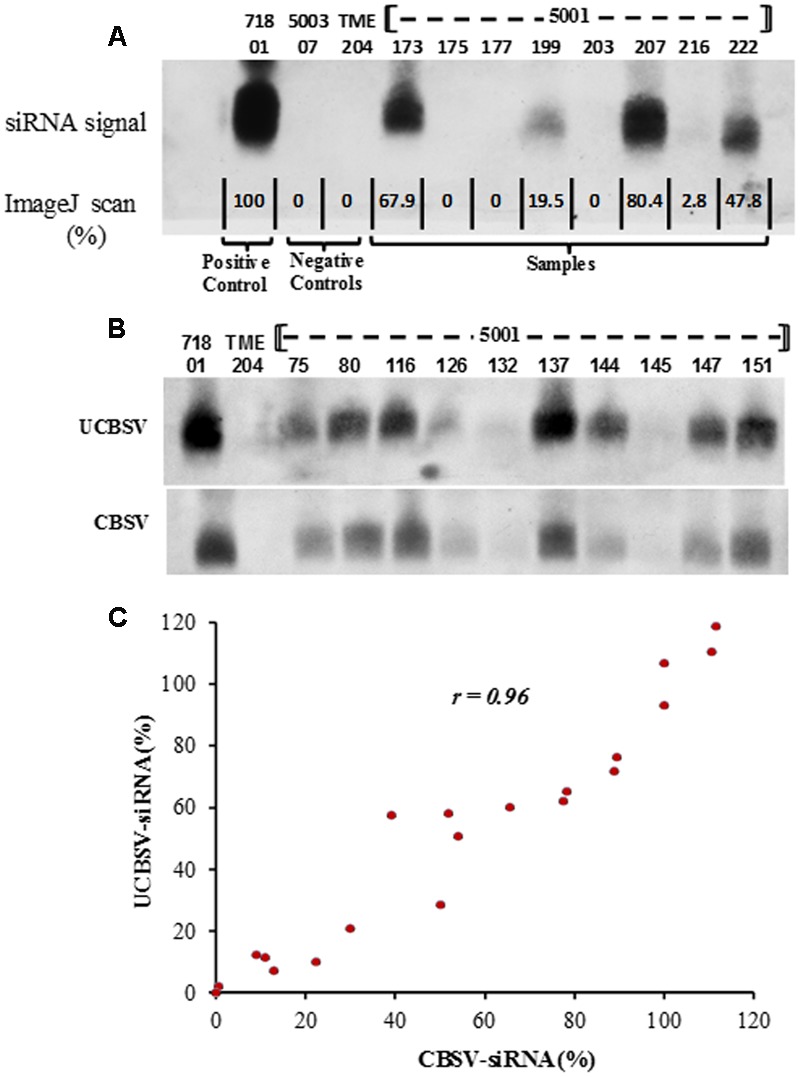
**Northern blot to determine expression of CP-derived siRNAs in transgenic p5001 plant lines of cassava cultivar TME 204.** p5001 plants harboring inverted repeats of the CP genes derived from CBSV and UCBSV were analyzed for presence of transgene-expressed siRNA using probes specific for the CP of each virus species. siRNA expression levels were quantified by assessing signal intensity with ImageJ software. **(A)** Expression of siRNAs specific to the CP of UCBSV. Control plant line 718-01 is included as a known high accumulator of UCBSV-CP specific siRNA **(B)** siRNA expression levels of p5001 lines assayed using siRNA probes for individual detection of UCBSV-CP and CBSV-CP with signal intensities expressed relative to known controls, **(C)** correlation between siRNA expression levels derived from UCBSV-CP and CBSV-CP within the same transgenic plant. siRNA expression levels are expressed as percent of high expressing lines included in each blot.

siRNA accumulation within *in vitro* leaves of transgenic p5001 TME 204 plants was assayed by blotting once with the CBSV-CP specific probe, and once with the UCBSV-CP specific probe. Signal intensity was quantified by scanning Northern blots and analysis with ImageJ software. All blots included positive control RNA extracted from plant line 718-01 of cultivar 60444 transgenic for construct p718 (Yadav et al., 2011). This plant line was previously shown to accumulate high levels of siRNAs against UCBSV, and to display effective resistance to CBSD under greenhouse and field conditions (Yadav et al., 2011; Ogwok et al., 2012). In addition, for quantification of siRNA derived from CBSV, plant line 5001-08 that accumulated levels of siRNA similar to 718-01 was used as a positive control in all blotting assays. Accumulation of CP-derived siRNAs was found to be variable across the transgenic p5001 lines tested. Examples of the signal intensities observed are shown in **Figure 2A**. These included: undetectable in lines 5001-175 and 5001-177; very low for line 5001-216 (2.8% of positive control levels); low in 5001-199 (20% of the positive control); and medium to high in lines p5001-22 (48% of the positive control), 5001-173 (68% of the positive control), and 5001-207 (80% of the positive control).

**FIGURE 3:**
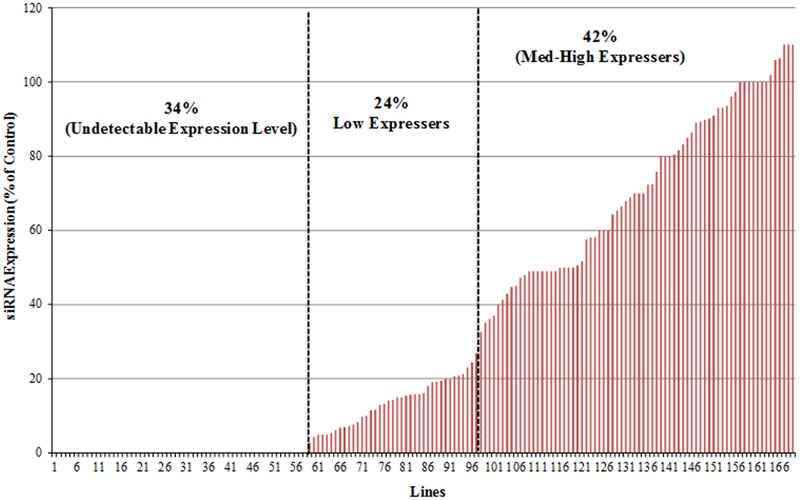
**Characterization of siRNA expression levels in 169 transgenic p5001 lines.** Transgenic TME 204 lines were generated using the p5001 construct that harbors inverted repeat near full-length sequences of CP genes from UCBSV and CBSV fused in tandem. Analysis of siRNA was performed by Northern blot of total RNA extracted from *in vitro* plantlets, using probes specific to each of the virus CP genes. siRNA signal intensities were quantified using ImageJ and compared to known control (set at 100%).

Analysis across all 169 p5001 lines revealed that 57 lines (34%) showed no detectable accumulation of siRNA, 40 lines (24%) showed relatively low expression level (1% -33% of the positive control) and 71 (42%) of the transgenic plant lines accumulated medium to high levels of siRNA (at ≥ 33.3% of the positive control) (**Figure 3**). Quantification of signal intensities (**Figures 2B,C**) indicated a strong positive correlation (*Pearson correlation, r* = 0.96) between siRNA accumulation levels specific to the CBSV-CP and UCBSV-CP sequences within the same transgenic plant line (**Figure 2C**). Therefore, for any given transgenic plant line, equivalent levels of siRNA accumulation were seen derived from the two CP sequence components present within the p5001 inverted repeat expression cassette.

### Resistance to Virus Challenge Correlates with Abundance of Transgene-Derived siRNA

**FIGURE 4:**
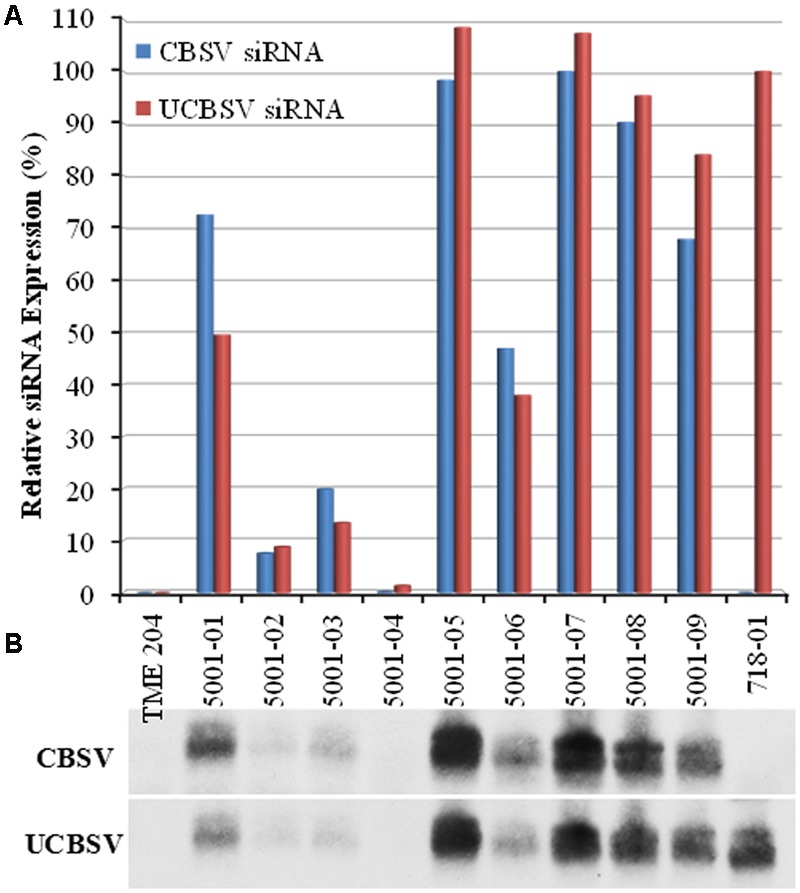
**Coat-protein-specific siRNA expression levels in p5001 transgenic plant lines used for challenge with CBSV. p5001 plant lines express an inverted repeat of CP genes derived from CBSV and UCBSV fused in tandem. (B)** siRNA expression levels detected by Northern blot, **(A)** scanned signal intensities quantified with ImageJ are presented relative to line 718-01 (UCBSV-CP) and line 5001-08 (for CBSV-CP specific siRNA). 718-01 plant line expresses an inverted of UCBSV-CP.

Efficacy of p5001 CP-derived siRNAs for generating resistance to CBSD was assessed by bud graft inoculation of greenhouse-grown TME 204 plants. Wild-type plants of cultivar 60444, confirmed to be infected with CBSV or UCBSV, were used as the source of inoculum (Wagaba et al., 2013). Experimental plants were challenged with the virulent Naliendele CBSV isolate (Mohammed et al., 2012) as a single virus challenge, and by dual challenge by grafting first with UCBSV followed 10–15 days later with CBSV as described previously by Wagaba et al. (2013). Three classes of p5001 plant types were tested. These included low siRNA accumulating lines (5001-02, 5001-03, 5001-04), medium accumulating lines (5001-01, 5001-06) and high accumulating lines (5001-05, 5001-07, 5001-08, and 5001-09) (**Figure 4**).

#### Resistance to Challenge with CBSV

When challenged with CBSV, CBSD leaf symptoms were first observed on non-transgenic TME 204 plants 7–15 days after graft inoculation. These were apparent as small chlorotic spots spread over the entire lamina of leaves about 10 nodes above the graft union (Wagaba et al., 2013), and subsequently by development of brown lesions on the stem. By 6 weeks after inoculation, 87.5 and 100% of the wild-type TME 204 plants showed leaf and stem symptoms, respectively (Supplementary Table 2).

Cassava brown streak disease symptom development on stems and leaves was negatively correlated with levels of transgenic CP-derived siRNAs. Fifty percent of the clonal replicates of plant line p5001-04 that accumulated the lowest levels of siRNA (**Figure 4**) developed CBSD symptoms on leaves and/or stems by 9 weeks after challenge. Plant lines 5001-02 and 5001-03 were also classed as low expressers of transgene-derived siRNAs but accumulated more than that seen in 5001-04 (**Figure 4**). No CBSD symptoms were observed on stems or leaves across 9–10 clonal replicates of plant lines 5001-02 or 5001-03 by 9 weeks after bud graft inoculation. These plants were then cut back to 5 nodes above the initial graft and CBSD symptom development assessed on the newly formed leaves. Thirty percent of clonal replicates from line 5001-02 and 22% of 5001-03 developed leaf and/or stem CBSD symptoms over the subsequent 3 weeks. Likewise, the medium level siRNA accumulating line 5001-06 developed no CBSD symptoms on shoot tissues by 9 weeks after graft inoculation, with 27% developing leaf, but no stem, symptoms after cutback at 12 weeks after graft inoculation. All plants of the medium-high siRNA accumulating lines 5001-01, 5001-05, 5001-07, 5001-08, and 5001-09 (**Figure 4**), remained free of CBSD symptoms before and after cutback throughout the 12-week observation period.

**FIGURE 5:**
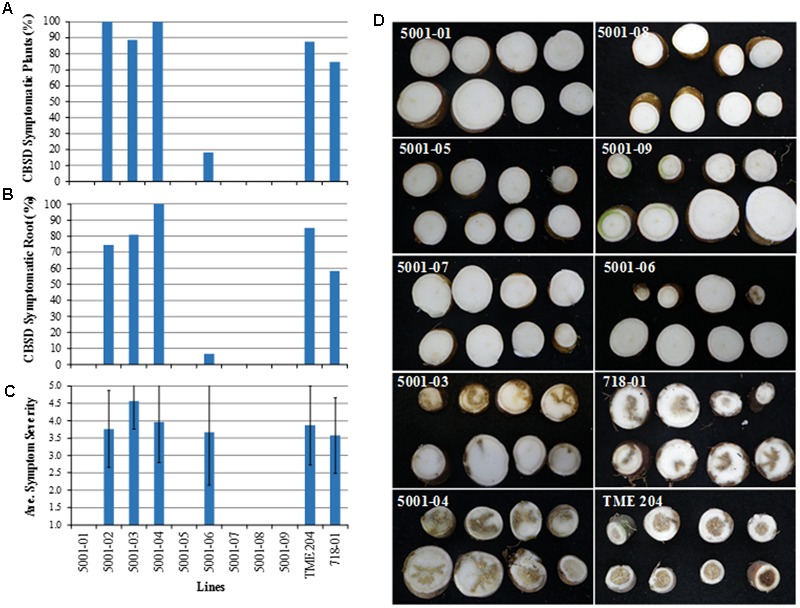
**Response of transgenic p5001 plant lines to CBSV challenge. p5001 plant lines express an inverted repeat of CP genes derived from UCBSV and CBSV fused in tandem.** Transgenic p5001 lines expressing different levels of CP-derived siRNAs were challenged with CBSV (isolate Naliendele CBSV[TZ:Nal3-1:07]) using chip bud graft method under greenhouse conditions. **(A)** number of plants per line showing CBSD symptoms in storage roots), **(B)** number of storage roots per line showing CBSD symptoms and **(C)** average CBSD symptom severity scores (scales 1–5). Bars show SD (*n* = 2–12). Experiments were repeated at least twice, **(D)** CBSD asymptomatic storage roots of transgenic plant lines 5001-01, 05, 07, 08, and 09 and symptomatic storage roots of lines 5001-03, 04, and 06, wild-type TME 204 and the control line 718-01. 718-01 plant line expresses an inverted repeat of UCBSV-CP.

Storage roots of plants inoculated with CBSV were harvested approximately 12 weeks after graft challenge. Each storage root was sliced transversely at 1–2 cm intervals along its length and evaluated for incidence and severity of CBSD necrotic symptoms. The proportion of clonal replicates per plant line showing presence of storage root CBSD symptoms ranged from 75 to 100% in the non-transgenic TME 204, and in control transgenic plants of cultivar 60444 line 718-01. Similar incidence of CBSD was seen in storage roots harvested from the low siRNA accumulating transgenic TME 204 lines 5001-02, 5001-03, and 5001-04 (**Figures 5A–D**). The frequency of storage roots within each line that displayed CBSD symptoms was also similar, ranging from 58–100% (**Figure 5B**). Conversely, 8% of clonal replicate plants of the medium siRNA accumulating line 5001-06 possessed storage roots displaying CBSD symptoms, with only three out of 45 roots harvested from this RNAi plant line showing root necrosis. Average CBSD symptom severity (scores 1–5) across all symptomatic lines was comparable and ranged from 3.6–4.6 (**Figure 5C**). As seen for shoot symptoms, all 146 storage roots harvested and examined from medium to high level siRNA expressing p5001 lines (5001-01, 5001-05, 5001-07, 5001-08, and 5001-09) were found to be asymptomatic for presence of CBSD (**Figures 5A–D**). Therefore, occurrence of CBSD storage root symptoms was strongly and inversely correlated with the levels of transgene CP-derived siRNA accumulation as determined before virus challenge (*Pearson correlation, r* = -0.94) (Supplementary Figure 1).

#### Resistance to Dual Challenge with CBSV and UCBSV

Resistance to CBSD imparted by the p5001 RNAi construct was further assessed by performing dual challenge with both virus species on a subset of the transgenic lines. This included two low (5001-03 and 5001-04), one medium (5001-06) and two high (5001-05 and 5001-07) siRNA accumulating events (**Figure 4**). Transgenic plants were first inoculated with an axillary bud obtained from source plants infected with UCBSV, followed 14 days later by graft inoculation with a bud infected with CBSV (Wagaba et al., 2013). Leaf and stem CBSD symptom development in dual graft challenges was similar to that seen for single CBSV challenge (Supplementary Table 3). In wild-type TME 204 plants, CBSD symptom development became apparent on leaves 2–3 weeks after virus inoculation. All (100%) control plants developed disease symptoms by 7.5 weeks after inoculation, with average symptom severities observed between 2.3 and 4.0 (scales of 1–5) (Supplementary Table 3). Transgenic plant lines 5001-03 and 5001-04 reached maximum CBSD incidences of 20 and 70%, respectively, on stem and/or leaves, with first symptom appearance delayed until 4–6 weeks after graft challenge. All plants of transgenic lines 5001-05, 5001-06, and 5001-07 remained CBSD symptom free on their shoot tissues.

**FIGURE 6:**
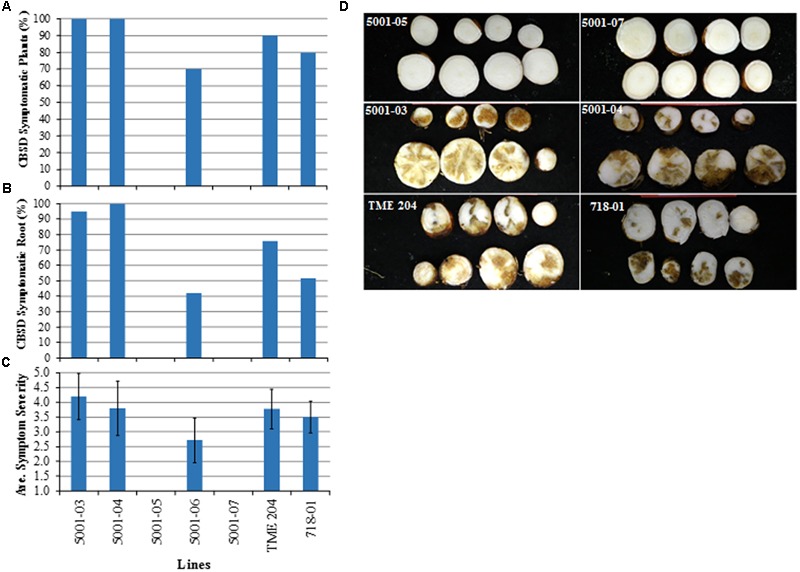
**Response of transgenic p5001 plant lines to dual challenge with CBSV and UCBSV. p5001 plant lines express an inverted repeat of CP genes derived from UCBSV and CBSV fused in tandem.** Plant line p718-01 expresses an inverted repeat of UCBSV-CP. Transgenic p5001 lines expressing different levels of siRNA were chip bud graft challenged first with UCBSV isolate UG:T04-42:04 followed 10–15 days later with CBSV isolate Naliendele TZ:Nal3-1:07 under greenhouse conditions. **(A)** number of plants per line showing CBSD symptoms in storage roots, **(B)** number of storage roots per plant line showing CBSD symptoms, and **(C)** average CBSD symptom severity scores (scales 1–5). Bars show SD (*n* = 7–10). Experiments were repeated at least twice, **(D)** asymptomatic storage roots of plant lines 5001-05 and 5001-07 and symptomatic storage roots of lines 5001-03, 04, wild-type TME 204 and the control line 718-01.

At 21 weeks after completion of dual graft challenge, plants were harvested and storage roots evaluated visually for CBSD. Presence of CBSD symptoms in storage roots followed a similar pattern to that seen on plants challenged with CBSV alone (**Figure 6**). All (100%) plants of the two low siRNA-expressing p5001 lines (5001-03 and 5001-04) showed CBSD storage root necrosis compared to 90% of non-transgenic TME 204 plants and 80% of 60444 line 718–801. In the medium siRNA-accumulating line 5001-06, 70% of the plants displayed CBSD storage root symptoms (**Figures 6A,D**). The number of storage roots within each plant line showing CBSD necrosis was 95–100% for 5001-03 and 5001-04, 42% in 5001-06, 52% in 718-01 and 76% for wild-type TME 204, with average CBSD severity scores of symptomatic lines varying from 2.7 to 4.2 (**Figures 6B,C**). In contrast, all plants of transgenic lines 5001-05 and 5001-07 were asymptomatic, with no disease observed across 20 (10 for each line) clonal replicates and a total of 60 harvested storage roots (**Figures 6A,D**).

### Detection of Virus in Graft Inoculated Plants

**FIGURE 7:**
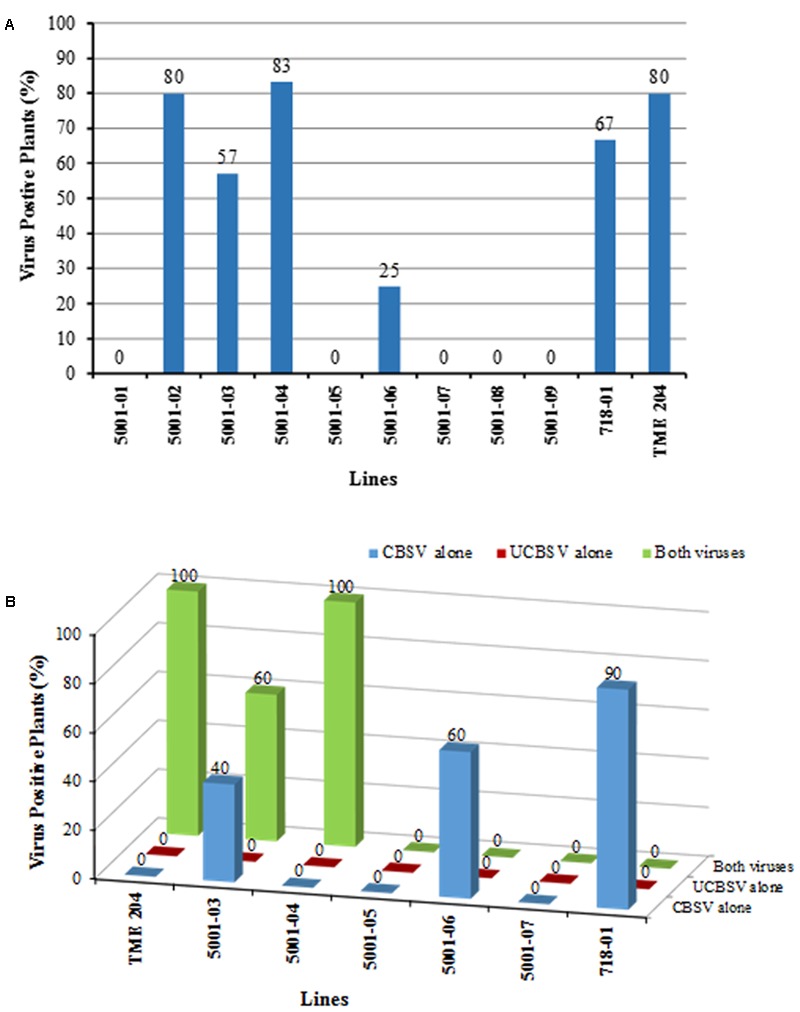
**Detection of cassava brown streak viruses in transgenic p5001 plant lines by RT-PCR. p5001 plant lines express an inverted repeat of CP genes derived from UCBSV-CP and CBSV-CP fused in tandem.** Plant line 718-01 expresses an inverted repeat of UCBSV-CP. **(A)** CBSV positive plants per line from symptomatic and asymptomatic leaf samples collected 9 weeks after chip bud graft inoculation with CBSV alone. **(B)** CBSV, UCBSV, or CBSV+UCBSV positive plants per line in transgenic p5001 lines from storage root samples collected at 22 weeks after grafting.

The youngest fully expanded leaves were sampled from plants challenged with CBSV at 9 weeks after bud grafting and assayed by RT-PCR for presence of the virus. Presence of CBSV was confirmed in 80% of non-transgenic TME 204 plants and 67% of 60444 control line 718-01. Within the low siRNA-accumulating lines (5001-02, 5001-03, and 5001-04), CBSV was detected in 80, 54, and 83% of the plants tested, respectively (**Figure 7A**). In the medium siRNA accumulating line 5001-06, only 25% of the clonal replicate plants tested positive for presence of CBSV, while all plants of the medium-high siRNA-accumulating lines (5001-01, 5001-05, 5001-07, 5001-08, and 5001-09) had no detectible CBSV in the sampled leaf tissues (**Figure 7A**).

Storage roots were analyzed by RT-PCR from plants co-inoculated with both CBSD causal pathogens using primers specific to the CP of each virus. All plants of wild-type non-transgenic TME 204 plants and the susceptible line 5001-04 were found to be positive for presence of both CBSV and UCBSV (**Figure 7B**). In the control line 718-01, 90% of the plants tested were positive for presence of CBSV, but all were found to be free of UCBSV. In the low siRNA accumulating line 5001-03, 60% of the clonal replicates were seen to be infected with both viruses, with the remaining 40% showing presence of CBSV only, while the medium level accumulating line 5001-06 had 60% of its plants positive for presence of CBSV, with the remaining plants free of detectable UCBSV or CBSV (**Figure 7B**). All plants of the transgenic lines 5001-05 and 5001-07 showed no detectable levels of CBSV and/or UCBSV within their storage root tissue (**Figure 7B**).

### Back-Inoculation to CBSD Susceptible Hosts

Back-inoculation experiments were performed in order to confirm whether resistant cassava plant lines were free from one or both viruses. Sap was extracted from cassava plants double-challenged with CBSV and UCBSV and used to inoculate *N. benthamiana*. Axillary buds from the same plant lines were bud grafted onto the CBSD susceptible cassava cultivar 60444. Infected plants of wild-type TME 204 and line 5001-04 were used as positive controls for bud grafting back to cultivar 60444.

**FIGURE 8:**
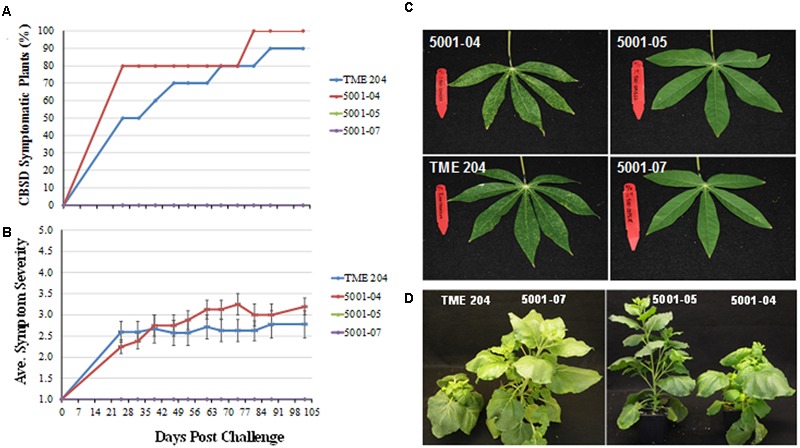
**Cassava brown streak disease symptom development in back-inoculated cassava and *Nicotiana benthamiana* plants.** Buds or leaves from transgenic p5001 plant lines and wild-type TME 204 plants inoculated with CBSV and UCBSV and asymptomatic TME 204 lines 5001-05 and 5001-07 and symptomatic 5001-04 and wild-type TME 204 plants were back inoculated to the susceptible cassava cultivar 60444 by chip bud grafting and to *N. benthamiana* by sap-inoculation. CBSD symptom development and severity was monitored on cassava for 14 weeks and for a period of 2–4 weeks on *N. benthamiana*. **(A)** Percent CBSD symptomatic 60444 cassava plants and **(B)** average severity score (scales of 1–5), **(C)** CBSD symptomatic (left) and asymptotic (right) plants inoculated with a bud derived from 5001-04 and TME 204, and with a bud derived from asymptomatic 5001-05 and 5001-07, respectively. **(D)** *N. benthamiana* plants inoculated with sap extracted from leaves obtained from the CBSD symptomatic TME 204 and transgenic 5001-04 and asymptomatic *N. benthamiana* plants sap-inoculated with leaf samples obtained from CBSD asymptomatic 5001-05 and 5001-07 cassava lines. p5001 plant lines express an inverted repeat of CP genes derived from UCBSV-CP and CBSV-CP fused in tandem.

Between 90 and 100% of 60444 plants inoculated from buds excised from infected donor plants developed CBSD symptoms on their leaves within 11–18 weeks (**Figures 8A–C**). In contrast, all 60444 plants graft inoculated with buds obtained from high siRNA-expressing lines 5001-05 and 5001-07 previously challenged with CBSV and UCBSV (**Figures 6** and **7**) remained free of CBSD symptom on leaves or stems (**Figures 8A–C**). Similarly, *N. benthamiana* plants inoculated with sap extracted from leaf samples prepared from high siRNA-accumulating and CBSD-asymptomatic lines 5001-05 and 5001-07 failed to develop leaf symptoms. This contrasted with plants inoculated with sap extracted from CBSD-challenged wild-type TME 204 and susceptible lines 5001-04. In this case, all *N. benthamiana* plants developed typical CBSD symptoms characterized by stunting and curled leaves within 2–4 weeks of inoculation (**Figure 8D**).

## Discussion

The farmer-preferred cultivar TME 204 possesses inherent resistance to CMD (Rabbi et al., 2014; Beyene et al., 2016) but is susceptible to CBSD (Ogwok et al., 2015). Transgenic TME 204 lines were genetically engineered with RNAi construct p5001 (Chauhan et al., 2015) with the goal of modifying this cultivar to generate TME 204 germplasm with robust resistance to CBSD and CMD. The p5001 construct described here builds on previous studies demonstrating control of CBSD by expression of an inverted repeat of the UCBSV-CP sequence (p718) (Yadav et al., 2011). Cassava plants transgenic for p718 expressing high levels of siRNA demonstrated resistance to the homologous virus species in the greenhouse (Yadav et al., 2011) and field (Ogwok et al., 2012). To ensure broad spectrum resistance against prevailing CBSD viruses, CP sequences from CBSV and UCBSV were combined within the p5001 construct. Data presented here confirms that p5001 transgenic plants are resistant to graft challenge with both CBSV and UCBSV under greenhouse conditions.

In this study, plant line 718-01, which displayed field-level resistance to both virus species (Ogwok et al., 2012; Odipio et al., 2014), succumbed to CBSV when bud-graft challenged under greenhouse conditions (**Figures 5** and **6**). RT-PCR analysis of the storage roots from plants of 718-01 co-inoculated with UCBSV and CBSV confirmed the presence of CBSV, but not UCBSV (**Figure 7B**), indicating that these plants remained resistant to the homologous virus species. Differences between field and greenhouse data demonstrated by line 718-01 could be explained by greater stringency of graft versus whitefly-mediated transmission of the ipomoviruses and/or the virulent nature of the CBSV Naliendele isolate (Mohammed et al., 2012) used in the present graft inoculations (**Figures 5** and **6**). Ogwok et al. (2015) also report differential resistance to CBSD pathogens in field-grown cassava plants, such that moderately resistant cultivars such as NASE 3, NASE 14, NASE 16, MH97/2961 and Nyaraboke were infected only with CBSV, not UCBSV. These cultivars are non-transgenic with unknown mechanism of tolerance to CBSD. In contrast, of the nine transgenic p5001 lines tested by bud graft inoculation, five lines (5001-01, 5001-05, 5001-07, 5001-08, and 5001-09) remained free of CBSD symptoms in both their shoots and storage root organs (**Figures 5** and **6**). Plants from all five lines were also found to be free of detectable levels of CBSV and UCBSV based on RT-PCR diagnosis (**Figure 7**). Absence of CBSD pathogens within these TME 204 transgenic plant lines was further demonstrated by back-inoculation to CBSD susceptible *N. benthamiana* and cassava plants (**Figure 8**).

Levels of resistance to CBSD were strongly and positively correlated with expression of CP-derived siRNA. The five transgenic lines that displayed resistance all accumulated medium-high levels of transgene-derived siRNA from both CP (**Figure 4**). This confirms previous findings for RNAi-mediated resistance to CBSD in *N. benthamiana* and cassava cultivar 60444 (Patil et al., 2011; Yadav et al., 2011). TME 204 lines transgenic for p5001 that accumulated low levels of siRNA (lines 5001-02, 5001-03, and 5001-04) were found to be infected with CBSV and UCBSV. Interestingly, line 5001-06 that accumulated a moderate level of siRNA showed resistance to UCBSV but not to CBSV (**Figures 4**–**6**) in a manner similar to line 718-01. The enhanced protection conferred by the p5001 construct compared to p718 suggests that siRNAs derived from CP genes representing the two virus species are significantly superior compared to UCBSV alone for imparting durable protection against CBSD.

Resources limit the number of plant lines that can be graft challenged under replicated experiments. However, by correlating levels of transgene-derived siRNAs with effective resistance to CBSD as reported here, it can be estimated that one third of the 169 p5001 transgenic plants screened by Northern blotting for siRNA accumulation (medium-high) would demonstrate effective resistance to CBSD. Absence of detectable siRNA expression in approximately one third of the lines (34%) and varying expression across the remaining transgenic p5001 TME 204 lines is most likely due to T-DNA integration position effects common for *Agrobacterium*-mediated plant transformation that affects transgene expression through epigenetic mechanisms such as DNA methylation (Kohli et al., 2010). Based on these findings, 25 transgenic p5001 TME 204 plant lines were selected, exported to NaCRRI, Namulonge, Uganda and KALRO, Mtwapa, coastal Kenya and established under confined field trial conditions. High levels of efficacy against vector-transmitted CBSD infection demonstrated by these plants are described in the accompanying report (Wagaba et al., 2017).

We previously reported unexpected loss of inherent CMD2-mediated resistance to cassava mosaic disease in transgenic p5001 lines of TME 204 (Beyene et al., 2016). The loss of resistance was not related to T-DNA integration or transgene-derived siRNA production. Instead, passage of tissues through somatic embryogenesis was sufficient to cause loss of CMD2-mediated resistance. Studies are underway to understand the genetic and molecular basis of the loss of resistance including a recently published genome-wide methylation map of CMD2-type cultivar TME 7 (Wang et al., 2015). Currently, work is ongoing to cross transgenic CBSD-resistant p5001 lines described here with wild-type CMD2-type cultivars in order to integrate both CMD and CBSD resistance in the same genetic background.

## Author Contributions

NT, CF, TA, DM, and GB conceived the project. GB, NT, HW, and MI designed the project, carried out the experiments, generated and analyzed the data. RC and NT generated transgenic plants. GB and NT wrote the manuscript. All contributed and agreed to the final content.

## Conflict of Interest Statement

The authors declare that the research was conducted in the absence of any commercial or financial relationships that could be construed as a potential conflict of interest.
