# Supplementary figures and images for: A Virus-Derived Stacked RNAi Construct Confers Robust Resistance to Cassava Brown Streak Disease
Source: Front Plant Sci. 2017 Jan 18;7:2052. doi: 10.3389/fpls.2016.02052 (PMC5241307; doi:10.3389/fpls.2016.02052)

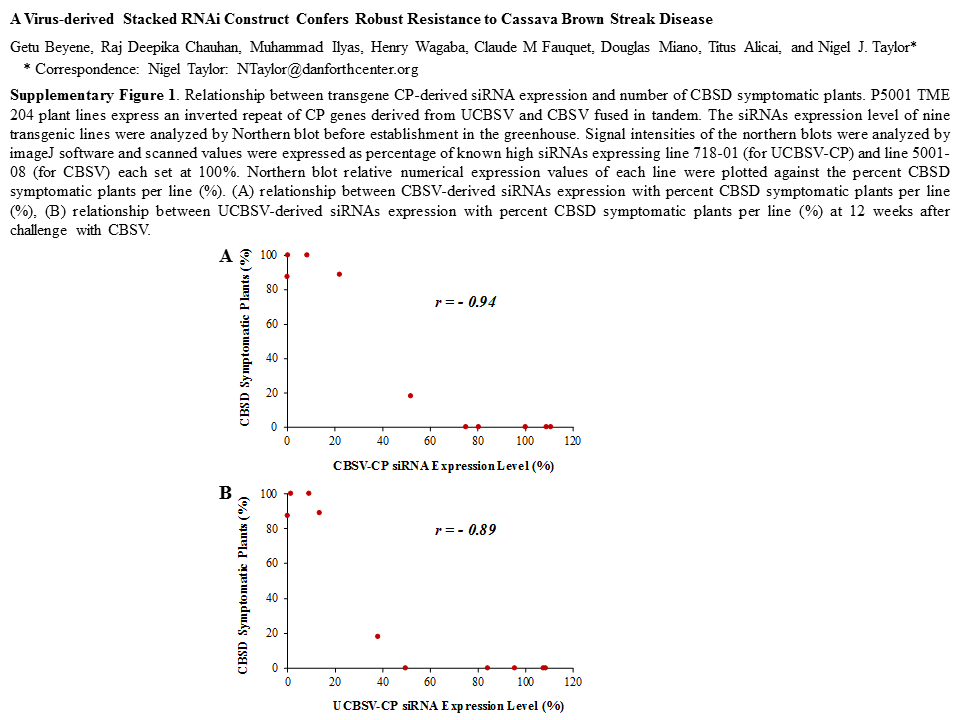

Supplement: Supplementary file 4 [file Image_1.tif]
